# Accidental allergic reactions to food in adolescents and adults: An overview of the factors involved and implications for prevention

**DOI:** 10.3389/falgy.2023.1062049

**Published:** 2023-03-10

**Authors:** Astrid Versluis, Thuy-My Le, Geert F. Houben, André C. Knulst, Harmieke Van Os-Medendorp

**Affiliations:** ^1^ Department of Dermatology and Allergology, University Medical Centre Utrecht, University Utrecht, Utrecht, Netherlands; ^2^ Center for Translational Immunology, University Medical Centre Utrecht, University Utrecht, Utrecht, Netherlands; ^3^ TNO, Netherlands Organization for Applied Scientific Research, Utrecht, Netherlands; ^4^ School of Health, Saxion University of Applied Sciences, Enschede, Netherlands

**Keywords:** food allergy, accidental allergic reactions, prevention, factors, accidental reactions

## Abstract

Accidental allergic reactions to food are one of the major problems in adult patients diagnosed with food allergy. Such reactions occur frequently, are often severe and are associated with higher medical and non-medical costs. The aim of this Perspective is to provide insight into the different factors involved in the occurrence of accidental allergic reactions and to present an overview of practical implications for effective preventive measures. Several factors affect the occurrence of accidental reactions. These factors are related to the patient, health care, or food. The most important patient-related factors are age, social barriers to disclosing their allergy and non-adherence to the elimination diet. With regards to healthcare, the degree to which clinical practice is tailored to the individual patient is an important factor. The major food-related factor is the absence of adequate precautionary allergen labeling (PAL) guidelines. Since many factors are involved in accidental allergic reactions, different preventive strategies are needed. It is highly recommended that health care be tailored to the individual patient, with regard to education about the elimination diet, support on behavioral and psychosocial aspects, usage of shared decision-making and taking into account health literacy. In addition, it is crucial that steps are taken to improve policies and guidelines for PAL.

## Introduction

Food allergy affects 0.3%–6% of the adults in Europe (1). At present, no curative treatment is available. The key interventions after diagnosing a food allergy are an elimination diet and emergency medication to treat accidental allergic reactions (2). Despite specific dietary advice, accidental reactions to food still occur in the daily life of food-allergic individuals (3, 4). A prospective study showed that approximately half of all food allergic adults experienced on average, two accidental reactions per year (4). The severity of accidental allergic reactions varies from mild to severe (3, 4), and is sometimes even fatal (3). Even with severe reactions, patients often fail to adequately use their emergency medication and do not always seek medical treatment (3, 4). This increases morbidity and the risk of fatal outcomes (5). In addition, accidental allergic reactions have a significant impact on costs: food allergic patients with accidental allergic reactions had sevenfold higher direct and indirect costs than food allergic patients without these reactions (6). In all subcategories (hospital admissions, primary care consultations, outpatient consultations, travel costs to healthcare facilities and sick leave costs due to accidental reactions), patients with accidental allergic reactions were shown to have higher costs than patients without accidental allergic reactions (6).

Given the impact of accidental allergic reactions on costs and their frequency and severity, increased prevention of these accidental reactions is needed. To be able to prevent accidental allergic reactions, it is important to identify factors that affect their occurrence. The aim of this Perspective is to give insight into the different factors involved in the occurrence of accidental allergic reactions and to provide an overview of practical implications for effective preventive measures.

## Many factors affect the occurrence of accidental allergic reactions

**Figure 1:**
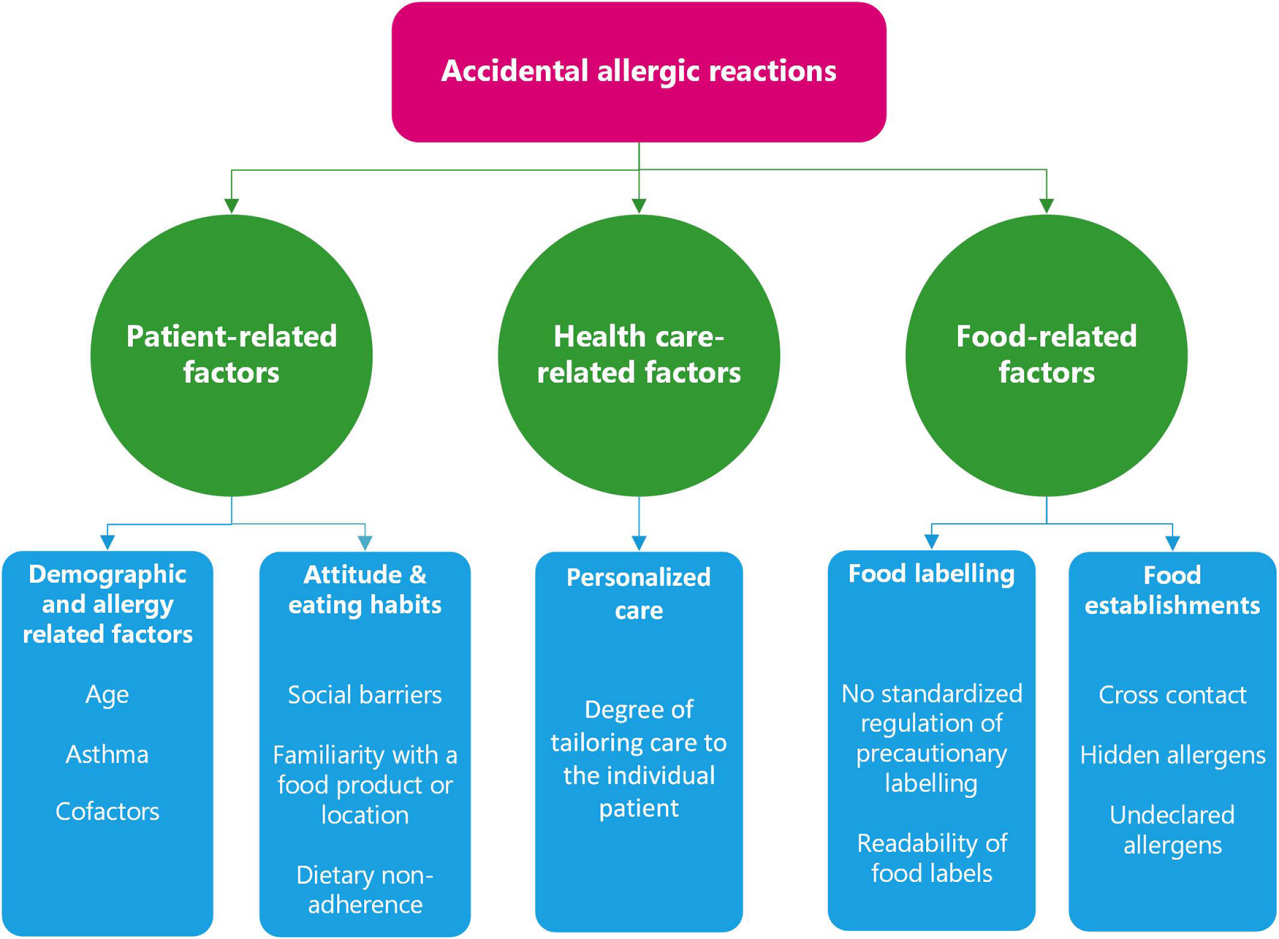
Factors affecting the occurrence of accidental allergic reactions.

Factors which influence the occurrence of accidental allergic reactions can be grouped into three categories: (1) patient-related, (2) health care-related and (3) food-related (Figure 1).

### Patient-related factors

Patient-related factors which influence the risk of accidental allergic reactions include patient age (7), history of asthma (2, 8), attitude and eating habits (3, 9), and cofactors (10–14) (Figure 1).

#### Age-related factors

Adolescents and young adults are at high risk of fatal reactions to food: among 32 fatalities, 69% occurred in patients between 13–21 years of age (7). This high percentage may be due to higher risk-taking behavior with regard to the management of the elimination diet by teenagers and adolescents (3). Adolescence is known as a period of heightened vulnerability to risk-taking behavior (15). Only 61% of adolescents always carry their adrenaline auto-injector, meaning that 39% do not (16). Adolescence is the period in which responsibility for dietary management transfers from the parents or carer to the patient. Specific strategies for this transition are important. Such strategies include: an early start of the transition process (11–13 years), using a multidisciplinary approach, discussing self-management in everyday contexts such as school/work and actively evaluating adherence (17).

#### Asthma

Food-allergic patients with asthma appear to be at higher risk of severe and even fatal reactions (2, 8). Of 16 fatalities in adult patients, all individuals for whom data was available had asthma (8). In a study about fatal and near fatal reactions to food in 13 children and adolescents, all had asthma, whereof 12 had asthma that was well controlled (18). The EAACI guidelines (2) recommend the use of an adrenaline auto-injector in food-allergic patients with persistent or severe asthma and short-acting beta agonists for every patient with co-existing asthma. Healthcare professionals should monitor asthmatic patients carefully, including training on when and how to treat reactions.

#### Attitude and eating habits: social barriers, familiarity with food/location and dietary change

Patients often experience social barriers to the disclosure of their allergy when eating outside their home, due to fear of potential social embarrassment (3). They may avoid situations that might lead to them being perceived as on overly fussy eater (19). It is important that patients communicate information about the food allergens they avoid, because then the person who prepares or provides the food can take this into account. Accidental reactions often (63%–74%) occur at locations outside the home (3, 20). In only 54% of the allergic reactions which occurred in restaurants had the customer informed restaurant staff of their food allergy (20). Healthcare professionals should therefore openly discuss social embarrassment with food allergic individuals, and emphasize the importance of disclosing their food allergy and associated restrictions when eating out.

Food-allergic consumers sometimes estimated the risk of eating a certain food product based on the type of food product or brand in combination with prior experiences, instead of reading the complete food label (3, 9). This approach may lead to individuals missing possible changes in ingredients.

Barriers to changing dietary behavior might play a role. A study which evaluated dietary adherence after 58 positive food challenges, showed that 52% of the patients followed a less strict diet than advised (9). Patients who were advised to change their diet following a positive food challenge, more often not adhered to the dietary advice compared to patients to whom no dietary change was advised (9). It has been shown in other chronic diseases that adherence to specific dietary patterns is challenging because of many barriers, such as higher costs, overall restrictive nature of the diet, social support and practical factors (21–23). Food-allergic individuals who were advised to strictly avoid the culprit food [meaning avoidance of the allergenic food and ingredients, including products with precautionary allergen labeling (PAL)] more often failed to adhere to dietary advice compared to patients who received less strict dietary advice (9). This might be explained by the fact that a strict elimination diet has more impact on food choices than a less strict diet. Another factor might be that many patients do not consider PAL credible and therefore ignore these labels (24, 25) (see paragraph *Food labeling issues are a main cause of accidental allergic reactions*). The high frequency of non-adherence to dietary advice, especially by patients advised to strictly avoid allergens, indicates that more guidance with regard to dietary behavior is needed.

#### The influence of cofactors on the threshold and severity of accidental allergic reactions seems to be limited

Cofactors are reported as factors which might influence the threshold and/or severity of allergic reactions in some patients. These include physical exercise, use of nonsteroidal anti-inflammatory drugs (NSAIDs), and consumption of alcohol (10, 11). A recent prospective study found that cofactors are often present during accidental allergic reactions (74%), but no significant correlation was found between the presence of cofactors and the severity of accidental allergic reactions (14). A number of studies found some, but only limited, influence of cofactors on the threshold and severity of allergic reactions to food (10, 11, 13). Recently, Dua et al. (12) published a prospective study in peanut-allergic patients who underwent three open peanut challenges: combined with exercise, combined with sleep deprivation, and with no intervention. This study reported that both sleep deprivation and exercise caused a reduction of the individuals’ threshold to peanut-related allergic reactions. Turner et al. (26) assessed the results of this study and concluded that this decrease is well within the intra-individual variability in reaction thresholds. Furthermore, the clinical center at which patients were evaluated had the largest impact on threshold variability (12, 26). Therefore, although cofactors can have some effect on threshold and severity of the reaction in some individuals, this does not appear to be any greater than the inherent shift in both clinical thresholds and risk of anaphylaxis identified in the wider food-allergic population, nor does it appear that such effects are predictable. The limited impact of cofactors is also indicated by the fact that population Eliciting Dose (ED) values calculated from reaction thresholds in the presence of the cofactors (12) were not lower than those based on the largest worldwide threshold dataset (27, 28), indicating that the variability caused by these cofactors does not exceed the population ED covered in this large threshold database. In conclusion, cofactors are frequently present in daily life, but the influence on thresholds and severity of accidental reactions seems to be limited. The exact influence and impact of cofactors on food-allergic reactions remains to be elucidated. Therefore, cofactors need to be considered when assessing a patient's medical history. If there is any suspicion of a role of cofactors in the occurrence of (severe) accidental reaction, it is advisable to inform patients about the possible role that cofactors can have in accidental reactions and how to manage these factors in daily life.

### Healthcare-related factors: a more patient-tailored approach is needed

While several patient-related factors can influence the occurrence of accidental allergic reactions, which factors are involved differ per patient. Therefore, in clinical practice, it is important that healthcare professionals investigate which factors apply for each individual. Based on the described patient-related factors, we formulated the following recommendations to tailor health care to the individual patient (Table 1):

1. **Education:** Education is a key intervention for management of food allergy and should cover allergen avoidance, symptoms recognition and when and how to treat reactions (2). It has been shown that patients do not always carry their self-injectable adrenaline and that self-injectable adrenaline had often not been immediately administered in fatal reactions (7, 18). Therefore, specific attention to the use of self-injectable adrenaline is important. The type of education materials can be tailored to the individual patient. Different tools can be combined, such as group sessions, written materials, mobile apps, video and online self-management programs (29–33). Furthermore, education of family and close relatives about potentially risky situations should be considered (2).
2. **Behavior change aspects:** It is important that support regarding behavioral aspects is tailored to the individual patient. For instance, specific attention and guidance is needed for patients who are advised to change their diet after diagnosis, and therefore need to change their dietary behavior. In these patients, extra follow-up consultations are important for supporting dietary and behavioral change (21). Furthermore, extra attention is needed for adolescents due to higher risk-taking behavior. Adolescents should be positively encouraged to self-manage their condition whilst still in a “semi-protected” environment, in preparation for adulthood (34). Specific attention to carrying the adrenaline auto-injector is important, for example by discussing the emotional effect of feeling different, offering strategies for carrying auto-injectors (e.g., purse) and emphasizing the importance of carrying the adrenaline auto-injector (16).
3. **Psychosocial aspects:** It is important to recognize psychosocial aspects like anxiety and social embarrassment, and tailor interventions to patients’ needs. Healthcare professionals can support patients with psychosocial aspects/issues by: (1) providing appropriate education *via* knowledgeable healthcare professionals about coping with the risks associated with allergen exposure, allergic symptoms and treatment of reactions, (2) enhancing self-efficacy, for instance by practicing assertiveness about avoidance of allergens, (3) assessing social concerns, for example discussing problems that patients and family encounter with regard to managing the food allergy and (4) meaning making, for instance by reflecting on the positive growth patients make due to the challenges of managing food allergy (35). Mental health professionals can help to address psychosocial concerns in case there is a greater need for support (36).
4. **Shared decision-making:** Shared decision-making is defined as a patient-centered approach wherein the healthcare professional and patient work together. It involves a mutual discussion about management and treatment options, which take into account the patient’s underlying preferences and values (37). This empowers patients to make decisions that they find most acceptable (37–39). Within the care for patients with food allergy, this can be applied, for instance, when deciding whether or not to undertake a food challenge or the type of tools to use in the education process.
5. **Health literacy:** Low health literacy is linked to poor health behavior and outcomes (43, 44). Approaches to address health literacy are: shared decision-making, using patient-friendly education materials (e.g., simple pictures, key points), using eHealth interventions (videos, interactive self-help tools), avoiding jargon, focusing on the key messages, repetition and using the “teach-back” method (ask the patient to recall what they have been told) (40–42).
6. **Multidisciplinary team:** A multidisciplinary team including allergists and/or immunologists, allergy-specialized dieticians, clinical nurse specialists, nurses and mental health professionals may provide and coordinate the healthcare regarding the needs of the individual patient (2, 36). It is important that each discipline attends to inter-professional collaboration and understands its own role and responsibilities and those of other team members (45, 46).

**Table 1:** Key points for a patient-centered approach.

Education	• Should cover allergen avoidance, symptoms recognition and when and how to treat reactions (2)
	• Should include specific attention to the use of self-injectable adrenaline (2)
	• The type of education materials should be tailored to the individual patient (29–33)
Behavior change aspects	• Support regarding behavioral aspects should be tailored to the patient • Should include extra attention and support for: (1) patients who are advised to change their diet, (2) adolescents, (3) carrying the adrenaline auto-injector (16, 21, 34)
Psychosocial aspects	• Recognition of psychosocial aspects like anxiety and social embarrassment, and tailor interventions to patients’ needs
	• Strategies to support psychosocial aspects: (1) appropriate education, (2) enhancing self-efficacy, (3) assessing social concerns, (4) support meaning making (35)
	• In case of major psychosocial concerns, support of a mental healthcare professional needs to be considered (36)
Shared decision-making	• Use shared decision-making to discuss management and treatment options (37–39)
Health literacy	• Address health literacy by: (1) shared decision-making, (2) patient-friendly educational materials, (3) e-health interventions, (4) focusing on the key messages, (5) repetition, (6) using the “teach-back” method (40–42)
Multidisciplinary team	• Use a multidisciplinary approach (including allergists and/or immunologists, allergy-specialized dieticians, clinical nurse specialists, nurses and mental health professionals) to provide and coordinate the healthcare regarding the needs of the individual patient (2, 36)

Applying the above-detailed recommendations will help to ensure that healthcare is better-tailored to the individual patient and contribute to decreasing the occurrence of accidental reactions. For future research, it would be interesting to evaluate the effectiveness of these recommendations with regard to the occurrence of accidental reactions.

### Food-related factors: better regulation of food labeling and food establishments needed

#### Food labeling issues are a main cause of accidental allergic reactions

A major issue affecting the occurrence of accidental allergic reactions, is the poor regulation regarding when and when not to use a PAL statement on prepackaged foods. PAL is used by manufacturers to give information about the possible occurrence of allergen contamination during the production process of food products. PAL is however poorly regulated and therefore is not always nor uniformly applied on food products. It is reported that 17%–68% of all manufactured foods contain PAL (47, 48). Chocolate, sweets and biscuits have such a description on more than 50% of the labels (49). Only 10% of prepackaged food products with a precautionary statement about peanuts had a detectable level of this allergen (50). This corroborates the notion that avoidance of products with PAL leads to major unnecessary dietary restrictions, with all their nutritional and social consequences. On the other hand, it was reported that there are prepackaged food products with clinically relevant levels of unlabeled allergens, which is a major concern because of the risk of severe accidental reactions (51, 52). In 37% of food products which caused accidental reactions, a non-ingredient allergen was detected (53).

Usage of PAL on prepackaged food products is often not based on a standardized risk assessment process. The current practice of deciding on when and how to use PAL has resulted in non-uniform application of PAL and generated more confusion and uncertainty than benefit for food allergic patients (54, 55). Many patients do not consider PAL credible and therefore ignore these labels (24, 25). Previously, several promising recommendations were given to improve these labels, including to only use PAL when there was a possibility that the allergen could be present at levels that might result in intakes in excess of a relevant reference dose (55, 56). Adoption of these recommendations will strongly contribute to global harmonization of risk-based allergen management and PAL. Educating food-allergic consumers (or those providing food for them, including food business operators) and other relevant stakeholders (e.g., risk assessors, healthcare providers) is critical, to ensure understanding of the applied principles and the implications of the chosen phraseology (55).

Issues with the readability of food labels also affect the occurrence of accidental reactions (3). It has been shown that less than 50% of patients considered allergy information to be clear (57). Furthermore, patients attributed different risk levels of unintended presence of allergens in prepackaged food products to different wordings of PAL, especially patients with higher levels of health literacy (57). There is a wide variety of ways in which allergen presence is currently communicated (58), which increases the risk of misinterpretation. Several recommendations have been proposed to improve allergen information on food labels including: ensuring readability of food information, presenting allergens in the ingredient list in bold, presenting topic order in a uniform manner on the label, providing an allergen information section, and using allergen icons (58). Moreover, it is recommended that there is a universally agreed upon uniform wording of PAL, that would unambiguously convey that the product is not suitable for individuals allergic to the specific allergen potentially contained in the product (55, 58). It is important that steps are taken to improve policies and guidelines, to translate such improvements into practice.

#### Food establishments

Miscommunication and poor knowledge of restaurant staff appears to be an important cause of increased risk of accidental allergic reactions. In food establishments, cross-contact can easily occur, for example by hands and cooking equipment (59). Hidden and undeclared allergens in menus may result in the occurrence of accidental reactions (20, 60). It is known that risks are higher in specific types of restaurants like Asian restaurants and ice cream shops (60). A study in the United States showed that only 73% of the servers correctly identified hen's egg as a major allergen and about 10% of managers and staff erroneously believed that a small amount of an allergen can be safely consumed by food allergic consumers (61). It can be assumed that this lack of knowledge leads to less careful working practices with regards to preventing cross-contact. A study in the United States showed that only 41% of the workers who primarily prepare or cook the food received food allergy training while working at their current restaurant (59). An American workgroup (60) has recommended measures for food establishments to lower cross-contact including educating staff that minimal cross-contact can cause allergic reactions, providing knowledge about cleaning methods to remove food allergens, creating a special allergen-free area in the kitchen and creating a separate pick-up area for allergen-free meals. However, several barriers are reported for implementation of food allergy training such as: high turnover of staff, lack of interest in food allergy training and high costs (60). Repeatedly offered food allergy training seems important to improve the knowledge of restaurant staff. To make this feasible, it seems necessary to reduce barriers, for example by making food allergy training easily and freely accessible by developing eLearnings or mobile apps.

## Conclusion

Accidental allergic reactions occur frequently and are associated with high costs. Their occurrence is affected by many factors, related to the patient, healthcare and food. The most important factors related to the patient are age-related, social barriers to disclosing their allergy and non-adherence to the elimination diet. With regard to healthcare-related factors, the degree to which clinical practice is tailored to the individual patient is important, especially with regard to education and support of behavioral and psychosocial aspects. A major food-related factor is absence of harmonized regulation regarding PAL. Therefore it is of great importance that steps are taken to improve policies and guidelines for PAL.

## Data Availability

The original contributions presented in the study are included in the article/Supplementary Material, further inquiries can be directed to the corresponding authors.
